# Geographic and socioeconomic diversity of food and nutrient intakes: a comparison of four European countries

**DOI:** 10.1007/s00394-018-1673-6

**Published:** 2018-03-28

**Authors:** Elly Mertens, Anneleen Kuijsten, Marcela Dofková, Lorenza Mistura, Laura D’Addezio, Aida Turrini, Carine Dubuisson, Sandra Favret, Sabrina Havard, Ellen Trolle, Pieter van’t Veer, Johanna M. Geleijnse

**Affiliations:** 1 Division of Human Nutrition, Wageningen University, PO Box 8129, 6700 EV Wageningen, The Netherlands; 2 Center for Health, Nutrition and Food, National Institute of Public Health, Brno, Czech Republic; 3 Council for Agricultural Research and Economics, Research Centre for Food and Nutrition, Via Ardeatina 546, 00178 Rome, Italy; 4 French Agency for Food, Environmental and Occupational Health and Safety (Anses)/Risk Assessment Department (DER), 14 rue Pierre et Marie Curie, 94701 Maisons-Alfort Cedex, France; 5 Division of Risk Assessment and Nutrition, National Food Institute, Technical University of Denmark, Søborg, Denmark

**Keywords:** Diet, Foods, Nutrients, Dietary guidelines, Europe, SUSFANS

## Abstract

**Purpose:**

Public health policies and actions increasingly acknowledge the climate burden of food consumption. The aim of this study is to describe dietary intakes across four European countries, as baseline for further research towards healthier and environmentally-friendlier diets for Europe.

**Methods:**

Individual-level dietary intake data in adults were obtained from nationally-representative surveys from Denmark and France using a 7-day diet record, Italy using a 3-day diet record, and Czech Republic using two replicates of a 24-h recall. Energy-standardised food and nutrient intakes were calculated for each subject from the mean of two randomly selected days.

**Results:**

There was clear geographical variability, with a between-country range for mean fruit intake from 118 to 199 g/day, for vegetables from 95 to 239 g/day, for fish from 12 to 45 g/day, for dairy from 129 to 302 g/day, for sweet beverages from 48 to 224 ml/day, and for alcohol from 8 to 15 g/day, with higher intakes in Italy for fruit, vegetables and fish, and in Denmark for dairy, sweet beverages and alcohol. In all countries, intakes were low for legumes (< 20 g/day), and nuts and seeds (< 5 g/day), but high for red and processed meat (> 80 g/day). Within countries, food intakes also varied by socio-economic factors such as age, gender, and educational level, but less pronounced by anthropometric factors such as overweight status. For nutrients, intakes were low for dietary fibre (15.8–19.4 g/day) and vitamin D (2.4–3.0 µg/day) in all countries, for potassium (2288–2938 mg/day) and magnesium (268–285 mg/day) except in Denmark, for vitamin E in Denmark (6.7 mg/day), and for folate in Czech Republic (212 µg/day).

**Conclusions:**

There is considerable variation in food and nutrient intakes across Europe, not only between, but also within countries. Individual-level dietary data provide insight into the heterogeneity of dietary habits beyond per capita food supply data, and this is crucial to balancing healthy and environmentally-friendly diets for European citizens.

**Electronic supplementary material:**

The online version of this article (10.1007/s00394-018-1673-6) contains supplementary material, which is available to authorized users.

## Introduction

Poor dietary habits are the second-leading risk factor for deaths and disability-adjusted life-years (DALYs) globally, accounting for 10.3 million deaths and 229.1 million DALYs in 2016 [1]. Low intakes of whole grains, fruit and vegetables, and nuts and seeds, and high intakes of alcohol and sodium ranked among the leading risk factors for early death and disability in European populations. However, as westernisation of diets progressed, diets high in red and processed meat, followed by diets high in sugar-sweetened beverages and low in milk are becoming a growing public health concern.

Dietary patterns are shaped by cultural, environmental, technological and economic factors, and they have become more similar over time owing to a general rise in living standards and globalisation of the food sector [2, 3]. Also in Europe there is a growing similarity of diets, in which traditional diets of Northern and Mediterranean countries are converging towards a more Western diet, viewed by the increased share of fruit and vegetables in Northern countries and the increased share of animal-based products in Mediterranean countries [4–6]. Increase in animal-based products and excessive caloric intake have been thought as a key factor in nutrition transition, which warrants the need for public health action to promote healthier food patterns consistent with traditional cultural preferences, hence the development of food-based dietary guidelines.

Food-based dietary guidelines are evidence-based integrated messages aimed at the general population for maintaining health and the prevention of non-communicable diseases [7, 8]. Promoting the intake of whole grains, fruit and vegetables, low-fat dairy and fish, and limiting the intake of red and processed meat, sugar-sweetened food products, alcohol and salt is covered by most national food-based dietary guidelines [9], although recommended quantities may differ. Monitoring food consumption patterns and assessing adherence to dietary guidelines in a nationally representative sample is especially regarded as a key instrument for evaluating the effectiveness of public health action towards a healthier diet.

In recent years, public health policies and actions have increasingly acknowledged the climate burden of food production and consumption, hence the need to address the food-climate connection, as outlined in the SUSFANS project (Metrics, Models and Foresight for European SUStainable Food And Nutrition Security) [10]. Production and technological changes in the food system will, however, not be sustainable without a change in food consumption patterns. The SUSFANS project, therefore, elaborates on the status-quo of diets and the design of optimised diets that are environmentally Sustainable, Healthy, Affordable, Reliable and Preferred (SHARP). This paper is a first step to study European food consumption patterns in terms of food groups and nutrients using national dietary survey data carried out at the individual level in four countries. Intakes of food groups and nutrients were compared with current food-based dietary guidelines and nutrient reference values, overall and in relevant population subgroups.

## Populations and methods

### Data sources

Individual-level dietary intake data from national dietary surveys representative for different European regions, i.e. Denmark (Scandinavia) [11], Czech Republic (Central East Europe) [12], Italy (Mediterranean) [13] and France (Western Europe) [14], were collated for adult population aged ≥ 18 years within the SUSFANS project [10]. These four countries were chosen to capture the wide range of foods and agricultural commodities, including their extreme intakes, that are incorporated in the diverse European food consumption patterns.

### Survey characteristics

Survey characteristics are shown in Table 1. National representativeness was ensured using random sampling based on civil registration systems in Denmark [11], national census data in Czech Republic [12] and France [14], and national census data with telephone books in Italy [13] that served as sampling frame, and followed by appropriate weighing for socio-demographic parameters, as applied in Denmark [11, 15] and France [14]. Surveys were organised throughout the whole year, covering the four seasons of the year, and have dietary data on week- and weekend-days.

**Table 1:** Dietary surveys in four European countries, i.e. Denmark, Czech Republic, Italy and France, including adult population only

	Denmark	Czech Republic	Italy	France
Survey characteristics, including adult population only				
Survey, year	The Danish National Survey on Diet and Physical Activity 2005–2008 National Food Institute, Technical University of Denmark (DTU)	Czech National Food Consumption Survey 2003–2004 (SISP04) National Institute of Public Health	Italian National Food Consumption Survey INRAN-SCAI 2005–2006 National institute for Research on Food and Nutrition	Individual and National Study on Food Consumption INCA-2 2006–2007 Agence Française de Sécurité Sanitaires des Aliments (AFSSA)
Population	18–75 years	18–90 years	18–98 years	18–79 years
Method of dietary assessment ^a^	7-day diet record on consecutive days	24-h recall on two non-consecutive days	3-day diet record on consecutive days	7-day diet record on consecutive days
Baseline characteristics of the study sample, including adult population only, *n* (%)				
Sample size (response rate)	2025 (54%)	1869 (54%)	2831 (33%)	2624 (60%)
Age, 18–64 years	1739 (85.9%)	1666 (89.1%)	2313 (81.7%)	2276 (86.7%)
Gender, men	777 (44.7%)	793 (47.6%)	1068 (46.2%)	936 (41.1%)
Educational level, low	248 (14.2%)	345 (20.7%)	692 (31.7%)	1039 (45.8%)
Overweight status, BMI ≥ 25	739 (43.2%)	864 (51.9%)	828 (35.8%)	871 (38.7%)

*BMI* Body Mass Index

^a^Included in the present study were for Czech Republic both day, for Denmark and France two randomly selected days, and for Italy the first and the last day of the national dietary survey

### Method of dietary assessment

In the four study countries, dietary intake was assessed over two to seven 24-h periods, either consecutively for 3–7 days using a diet record, as applied in Denmark, Italy and France [11, 13, 14], or non-consecutively spaced over a 3–5 months sampling period using two replicates of 24-h recall, as applied in Czech Republic [12]. In the present analyses, dietary intake from two random days has been reported. To this end, two non-consecutive days were sampled in Denmark, Italy and France, whereas all available days were used in Czech Republic.

### Food and nutrient intakes

Intakes of food groups and nutrients were calculated for each subject from the mean of the selected two days, and were standardised for energy using the density method. Densities were calculated as the absolute value divided by total energy intake, and multiplied by 2000 kcal. Harmonised food groups, including similar foods, have been elaborated using the ‘Exposure Hierarchy’ of the food classification and description system FoodEx2 developed and revised in 2015 by the European Food Safety Authority (EFSA) [16, 17]. A main challenge to encounter when grouping the foods was the level of food disaggregation; disaggregation of foods into ingredients was only considered as necessary for composite/prepared foods provided that the food itself was not included in FoodEx2, but its ingredients are. Nutrient intakes were calculated from dietary sources only, i.e. excluding dietary supplements, using country-specific food composition tables [18–24]. Intakes of added sugar, plant and animal protein were calculated based on food selection. Added sugar was defined as the total sugar intake minus sugars naturally occurring in fruits, vegetables and dairy. Plant protein was defined as protein derived from cereals, legumes, nuts and seeds, and others (including potatoes, vegetables, fruits, etc.). Animal protein was defined as protein derived from meat and meat products, fish and fish products, egg and egg products, milk and milk products (including cream, cheese and butter). None of the data excluded under- and over-reporting, however, misreporting was identified using Goldberg equation [25] and adopted by Black [26] (Online Resource 1).

## Dietary quality

### Foods

To evaluate European populations’ energy-standardised food group intakes, references values were set for the food groups that are important for disease risk reduction based on an inventory of the current food-based dietary guidelines of European countries. Minimum values were set for foods that are beneficial for health, such as fruits and vegetables, and maximum values for foods that are unfavourable for health, such as red and processed meat (see Box 1). Reference values were derived using the 2015 Dutch food-based dietary guidelines [8] as reference point, complemented by the food-based dietary guidelines of the four countries [27–30] in which the less restrictive reference values were chosen.

**Box 1:** A set of food-based dietary guidelines for European countries, including their exposure definition and reference values, developed for the SUSFANS project

	Exposure definition	Reference values^a^
Foods to increase		
Fruit	All kind of fruits (including fresh, dried, tinned or canned fruit products, but excluding fruit juice)	≥ 200 g/day
Vegetables	All kind of vegetables (including fresh, dried, tinned or canned vegetable products, but excluding potatoes, vegetable juices and vegetables from soup, sauces and ready-to-eat products)	≥ 200 g/day
Legumes	Kidney beans, pinto beans, white beans, black beans, garbanzo beans (chickpeas), lima beans, split peas, lentils, and edamame (green soybeans)	≥ 135 g/week (≥ 19 g/day)
Nuts and seeds	Walnuts, almonds, hazel, cashew, pistachio, macadamia, Brazil, pecan, pine nuts, flax seeds, sesame seeds, sunflower seeds, pumpkin seeds, poppy seeds, and peanut	≥ 15 g/day
Dairy products	Food products produced from the milk of mammals, including milk, yoghurt, fresh uncured cheese, quark, custard, milk puddings, excluding cheese and butter	≥ 300 g/day
Fish	All kind of fish and fish products	≥ 150 g/week (≥ 21 g/day)
Foods to decrease		
Red and processed meat	Red meat: all mammalian muscle meat, including beef, veal, pork, lamb, mutton, horse and goat, excluding rabbit meat; Processed meat: meat transformed through salting, curing, fermentations, smoking or other processed to enhance flavour or improve preservation (e.g. meat products as sandwich filling, ready-to-eat minced meat, sausages, etc.)	≤ 500 g/week (≤ 71 g/day)
Cheese	All types of cheese formed by coagulation of milk protein casein	≤ 150 g/week (≤ 21 g/day)
Sugar-sweetened beverages	Cold beverages with added sugars (sucrose, fructose or glucose), for example fruit juices, fruit nectars, soft drinks, ice teas, vitamin-water or sports drinks with added sugars	≤ 500 ml/week (≤ 71 ml/day)
A lcohol (Ethanol)	Ethanol content calculated from all kind of alcoholic beverages	≤ 10 g/day
Foods to replace^b^		
Whole grains	Whole grains (bran, germ and endosperm in their natural proportion) from cereals, pasta, bread, breakfast cereals and other grain sources	Replace white grains by whole grains
White meat	Meat from all kind of poultry, including rabbit meat	Replace red and processed meat by white meat
Soft margarine and oils	Soft margarine: soft-solid fats made from vegetables oils; Oils: liquid fats at room temperature derived from plants or fish	Replace butter and hard margarines by soft margarine and oils

^a^Reference values were derived from current food-based dietary guidelines, using the 2015 Dutch food-based dietary guidelines [8] as reference point, complemented by the food-based dietary guidelines of the four countries [34–37] in which the less restrictive reference values was chosen (Quantitative guideline)

^b‘^Foods to replace’ represent food groups for which insufficient convincing evidence was available to set a fixed cut-off point, however replacement of those food products by a healthier alternative is recommended (Qualitative guideline)

### Nutrients

To evaluate European populations’ energy-standardised nutrient intakes, nutrient density of the diet was quantified using Nutrient Rich Diet (NRD) score [31, 32], i.e. overall summary estimate of nutrient intakes based on the principles of the Nutrient Rich Food Index [33, 34]. The NRD algorithm was calculated as:

$${\text{NRD}}~X \cdot Y=~\mathop \sum \limits_{i}^{{i=X}} \frac{{{Q_{{\text{nutrient}}~i}}}}{{{\text{DR}}{{\text{V}}_i}}} \times 100 - ~\mathop \sum \limits_{j}^{{j=Y}} \frac{{{Q_{{\text{nutrient}}~j}}}}{{{\text{MR}}{{\text{V}}_j}}} \times 100$$

where *X* is the number of qualifying nutrients, *Y* is the number of disqualifying nutrients, *Q* nutrient *i* or *j* is the average daily intake of nutrient *i* or *j*, DRV is the dietary reference value of qualifying nutrient *i* and MRV *j* is the maximum recommended value of the nutrient to limit *j*. DRVs are defined using reference values from EFSA [35], i.e. average requirement (AR), and adequate intake (AI) if AR cannot be set, and MRVs using reference values of World Health Organisation [36, 37] and Food and Agriculture Organisation [38].

In the present analyses, NRD9.3 and NRD15.3 were used. The NRD9.3, including nine nutrients for which intake should be promoted (protein, dietary fibre, calcium, iron, potassium, magnesium, and vitamin A, C and E) and three nutrients for which intake should be limited (saturated fat (SFA), added sugar, and sodium), standardised for 2000 kcal/day diet and capped nutrient intake at 100% of DRV was primarily chosen, based on its validation among US populations [33, 34]. To capture more nutrients that are potentially relevant for European populations, we also used its extended version, i.e. NRD15.3 that additionally included mono-unsaturated fatty acids, zinc, vitamin D and B-vitamins (B1, B2, B12, folate), but excluded magnesium. A sub-score on the intake of qualifying nutrients is represented in NRD9 and NRD15, and that of disqualifying nutrients in NRDX.3, while the total score, i.e. NRD9.3 and NRD15.3, is a combination of both.

### Estimating the dietary quality of European populations’ diets

Percentages of the population that adhere to food-based dietary guidelines and percentages of the population with inadequate nutrient intakes were estimated using the AR cut-point method [39], without correction for within subject variability. This percentage would be interpreted as proxy figures for adherence and inadequacy, because of different survey’s methodologies. When the DRV of the nutrient under study was defined as an AI (dietary fibre, potassium, magnesium, vitamin D, E and B12), this percentage of populations with intake below AI was only applicable for comparison between countries and population subgroups. Dietary intakes were characterised in the overall country-specific population of adults aged ≥ 18 years and in relevant population subgroups by age, gender, educational level, and overweight status. Subgroups by age included younger and middle-aged adults (18–64 years) and elderly (≥ 65 years). Younger and middle-aged adult populations were additionally stratified by gender, educational level using three categories, i.e. primary or lower secondary degree (‘low’), higher secondary degree (‘intermediate’) and university or post-university degree (‘high’), and overweight status using two categories, i.e. BMI < 25 and ≥ 25 kg/m^2^.

As the information available consisted only of summarised data (i.e. mean and standard deviation of the energy-standardised dietary intake under study and sample size), analysis of variance test was performed to check whether there were differences in mean intake of food groups and nutrients between countries and within countries by population subgroups of age, gender, educational level and overweight status. Bonferroni post hoc test was used for multiple comparisons. A two sided *p* value below 0.0001 was considered as statistically significant. Statistical analyses were performed with SAS version 9.3 (SAS Institute Inc.).

## Results

### Baseline characteristics

Age and gender distribution were comparable between countries, with 80–90% of the population aged 18–64 years and 40–48% being men. Distribution of educational level varied markedly between countries; a low proportion of low-educated subjects in Denmark (15%) and a high proportion in France (46%); but proportion of the high-educated subjects was the lowest in Czech Republic (8%) and varied between 23–33% for Denmark, Italy and France. Approximately half of the Czech population (52%) was overweight, BMI ≥ 25 kg/m^2^, whereas overweight in Denmark (44%), France (39%) and Italy (36%) was less prevalent.

### Foods

Table 2 shows the energy-standardised intakes of food groups and general adherence to food-based dietary guidelines in four European adult populations, aged ≥ 18 years. Stratified intakes by age, gender, educational level and overweight status are shown in Table 3.

**Table 2:** Energy-standardised food group intakes and the adherence to their corresponding food-based dietary guidelines in four European populations, aged ≥ 18 years

	Cut-offs	Denmark (*n* = 2025)				Czech Republic (*n* = 1869)				Italy (*n* = 2831)				France (*n* = 2624)			
		Mean	Median	(P25; P75)	%adh	Mean	Median	(P25; P75)	%adh	Mean	Median	(P25; P75)	%adh	Mean	Median	(P25; P75)	%adh
Foods to increase																	
Fruit, g/day	≥ 200	174*	133	(36.0; 255)	35%	118*	83	(12.0; 171)	20%	199*	163	(76; 275)	40%	140*	95	(0.0; 210)	26%
Vegetables, g/day	≥ 200	147*	112	(63; 184)	21%	95*	74	(39.0; 127)	10%	239*	206	(138; 300)	53%	187*	157	(84; 254)	37%
Legumes. g/day	≥ 19	6.5	1.6	(0.0; 6.7)	10%	7.5	0.0	(0.0; 3.0)	12%	11.0	0.0	(0.0; 2.4)	19%	16.5*	0.0	(0.0; 0.8)	18%
Nuts and seeds, g/day	≥ 15	2.2	0.0	(0.0; 0.0)	5%	2.6	0.0	(0.0; 0.0)	7%	0.5*	0.0	(0.0; 0.0)	1%	1.7	0.0	(0.0; 0.0)	3%
Dairy products, g/day	≥ 300	302*	248	(113; 422)	41%	134	94	(31.0; 192)	12%	129	116	(8.0; 20)	8%	199*	152	(55; 290)	24%
Fish, g/day	≥ 21	18.0	5.5	(0.0; 24.1)	28%	11.7	0.0	(0.0; 0.0)	17%	44.6*	6.5	(0.0; 77)	42%	34.3*	4.3	(0.0; 54)	43%
Foods to decrease																	
Red and processed meat, g/day	≤ 71	94	85	(51; 127)	39%	88	82	(46.0; 125)	42%	84	77	(39.2; 119)	51%	93	82	(40.5; 133)	43%
Cheese, g/day	≤ 21	29.3	24.3	(11.3; 42.0)	44%	20.9*	13.2	(0.0; 33.0)	63%	53*	47.2	(16.2; 76)	28%	30.1	24.0	(2.9; 45.6)	46%
Sweet beverages^a^, ml/day	≤ 71	224*	127	(0.0; 305)	40%	108	0.0	(0.0; 144)	63%	47.5*	0.0	(0.0; 65)	76%	121	6.0	(0.0; 171)	56%
Alcohol (ethanol), g/day	≤ 10	14.6*	7.3	(0.0; 22.6)	56%	10.3	4.4	(0.0; 16.0)	66%	8.2	0.1	(0.0; 13.7)	67%	9.3	0.1	(0.0; 14.5)	67%
Foods to replace																	
Cereals, total, g/day	–	26.1*	16.9	(6.7; 35.0)	–	48.2	32.5	(11.0; 72)	–	46.6	38.3	(0.6; 73)	–	38.8*	16.05	(0.0; 57)	–
Cereals, whole grains, g/day	–	0.4	0.0	(0.0; 0.0)	–	0.1	0.0	(0.0; 0.0)	–	0.8	0.0	(0.0; 0.0)	–	1.8	0.0	(0.0; 0.0)	–
Pasta, total, g/day	–	5.2*	0.0	(0.0; 1.2)	–	39.9*	13.6	(0.0; 66)	–	52*	48.4	(29.8; 82)	–	10.3*	0.0	(0.0; 0.0)	–
Pasta, whole grains, g/day	–	–	–		–	0.0*	0.0	(0.0; 0.0)	–	0.3*	0.0	(0.0; 0.0)	–	9.8*	0.0	(0.0; 0.0)	–
Bread, total, g/day	–	149*	140	(94; 194)	–	122*	118	(83; 157)	–	109*	103	(60; 151)	–	98*	92	(51; 139)	–
Bread, whole grains, g/day	–	52*	44.3	(22.4; 72)	–	7.9*	0.0	(0.0; 0.0)	–	41.4*	0.0	(0.0; 70)	–	16.3*	0.0	(0.0; 6.1)	–
Breakfast cereals, total, g/day	–	11.8*	0.6	(0.0; 18.0)	–	2.9	0.0	(0.0; 0.0)	–	1.5	0.0	(0.0; 0.0)	–	5.3*	0.0	(0.0; 0.0)	–
Breakfast cereals, whole grains, g/day	–	9.3*	0.0	(0.0; 12.1)	–	1.9*	0.0	(0.0; 0.0)	–	0.5*	0.0	(0.0; 0.0)	–	3.4*	0.0	(0.0; 0.0)	–
Red meat, g/day	–	66*	57.1	(28.3; 93)	–	34.0*	28.4	(0.0; 55)	–	58	53	(0.0; 89)	–	58	45.6	(0.0; 91)	–
Processed meat, g/day	–	27.3	19.4	(7.1; 37.2)	–	54*	44.5	(14.0; 80)	–	25.5	19.4	(0.0; 38.9)	–	34.7*	22.6	(0.0; 54)	–
White meat, g/day	–	21.3	1.6	(0.0; 29.9)	–	22.5	0.0	(0.0; 41.0)	–	23.5	0.0	(0.0; 44.9)	–	31.5*	0.0	(0.0; 52)	–
Butter and hard margarines, g/day	–	24.8*	22.7	(13.5; 33.8)	–	17.6*	15.5	(7.0; 25.0)	–	2.8*	0.0	(0.0; 3.8)	–	16.3*	13.7	(5.8; 24.0)	–
Soft margarine and oils, g/day	–	1.9*	0.0	(0.0; 1.5)	–	15.0*	13.1	(7.0; 21.0)	–	34.8*	34.0	(26.3; 42.7)	–	11.2*	7.4	(0.4; 17.3)	–

Intake of food groups are standardised to a 2000 kcal/day diet

%adherence represents a proxy for the percentage of the population that adhere to food-based dietary guidelines

^a^Sweet beverages instead of sugar-sweetened beverages due to a lack of detailed data on beverages

*Bonferroni *p* < 0.0001 test comparison for intake that was significantly different from all other three countries under study

**Table 3:** Energy-standardised food group intakes and the adherence to their corresponding food-based dietary guidelines in four European populations in subgroups by age, gender, educational level, and overweight status: main findings

	Cut-offs	Subgroups by age									Subgroups by gender^a^								
		Younger and middle-aged adults				Elderly, ≥ 65 years				*p* value	Men				Women				*p* value
		Mean	Median	(P25; P75)	%adh	Mean	Median	(P25; P75)	%adh		Mean	Median	(P25; P75)	%adh	Mean	Median	(P25; P75)	%adh	
Denmark		(*n* = 1739)				(*n* = 286)					(*n* = 777)				(*n* = 962)				
Fruit, g/day	≥ 200	171	126	(32.2; 251)	34%	197	159	(81; 281)	40%	0.011	120	74	(0.5; 172)	21%	222	187	(74; 324)	47%	< 0.0001
Vegetables, g/day	≥ 200	151	114	(64; 189)	22%	119	98	(54; 167)	16%	< 0.0001	117	95	(54; 146)	13%	185	141	(84; 231)	31%	< 0.0001
Legumes, g/day	≥ 19	6.6	1.8	(0.0; 7.1)	10%	5.3	0.9	(0.0; 4.6)	10%	< 0.0001	5.9	1.3	(0.0; 5.6)	8%	7.3	2.2	(0.0; 8.6)	11%	< 0.0001
Red and processed meat, g/day	≤ 71	95	87	(52; 128)	38%	83	73	(41.5; 108)	48%	0.001	109	100	(66; 143)	29%	82	75	(43.3; 114)	47%	< 0.0001
Alcohol, g/day	≤ 10	13.8	6.4	(0.0; 21.5)	58%	20.5	15.0	(1.7; 29.8)	40%	< 0.0001	16.6	10.0	(0.0; 25.6)	50%	10.9	0.0	(0.0; 17.0)	66%	< 0.0001
Czech Republic		(*n* = 1666)				(*n* = 203)					(*n* = 793)				(*n* = 873)				
Fruit, g/day	≥ 200	115	79	(10.0; 167)	19%	143	118	(38.7; 218)	28%	0.006	66	39	(0.7; 93)	6%	160	128	(51; 224)	31%	< 0.0001
Vegetables, g/day	≥ 200	95	75	(39.3; 128)	10%	94	70	(39.4; 122)	8%	0.874	78	61	(35.0; 106)	5%	111	87	(46.0; 151)	14%	< 0.0001
Legumes, g/day	≥ 19	7.6	0.0	(0.0; 2.2)	11%	6.7	0.0	(0.0; 4.2)	13%	0.591	6.1	0.0	(0.0; 1.7)	10%	9.0	0.0	(0.0; 2.6)	12%	0.012
Red and processed meat, g/day	≤ 71	89	81	(44.8; 125)	42%	83	79	(45.3; 118)	42%	0.253	108	103	(69; 142)	27%	71	64	(28.4; 103)	55%	< 0.0001
Alcohol, g/day	≤ 10	10.7	5.1	(0.0; 17.0)	65%	7.4	0.0	(0.0; 9.4)	77%	0.002	15.8	12.5	(1.2; 23.5)	47%	6.1	0.0	(0.0; 8.6)	81%	< 0.0001
Italy		(*n* = 2313)				(*n* = 518)					(*n* = 1068)				(*n* = 1245)				
Fruit, g/day	≥ 200	185	153	(67; 257)	37%	257	222	(125; 333)	54%	< 0.0001	153	125	(50.4; 220)	28%	214	185	(88; 292)	45%	< 0.0001
Vegetables, g/day	≥ 200	238	205	(134; 299)	52%	241	215	(149; 307)	55%	0.680	222	190	(126; 282)	47%	252	156	(145; 317)	56%	< 0.0001
Legumes, g/day	≥ 19	10.7	0.0	(0.0; 2.9)	19%	12.4	0.0	(0.0; 0. 0)	19%	0.194	10.1	0.0	(0.0; 3.9)	19%	11.3	27.1	(0.0; 2.3)	19%	0.265
Red and processed meat, g/day	≤ 71	85	77	(37.6; 120)	65%	75	68	(31.6; 111)	62%	0.015	88	81	(43.6; 122)	65%	82	74	(32.7; 119)	64%	< 0.0001
Alcohol, g/day	≤ 10	7.8	0.1	(0.0; 12.7)	70%	10.0	2.6	(0.0; 16.5)	60%	0.0002	11.3	6.8	(0.0; 18.9)	57%	4.8	8.4	(0.0; 7.0)	80%	< 0.0001
France		(*n* = 2276)				(*n* = 348)					(*n* = 936)				(*n* = 1340)				
Fruit, g/day	≥ 200	129	77	(0.0 ; 198)	23%	209	174	(77; 309)	42%	< 0.0001	103	65	(0.0; 154)	17%	148	103	(0.0; 219)	28%	< 0.0001
Vegetables, g/day	≥ 200	182	152	(80; 248)	36%	219	196	(110; 293)	46%	< 0.0001	152	128	(65; 204)	26%	202	173	(95; 272)	45%	< 0.0001
Legumes, g/day	≥ 19	15.9	0.0	(0.0 ; 0.8)	17%	20.9	0.0	(0.0; 5.3)	20%	0.040	17.7	0.0	(0.0; 1.8)	19%	14.6	0.0	(0.0; 0.4)	16%	0.068
Red and processed meat, g/day	≤ 71	94	84	(40.7; 134)	43%	90	79	(37.8; 133)	45%	0.316	101	92	(49.8; 143)	38%	88	77	(33.9; 127)	47%	< 0.0001
Alcohol, g/day	≤ 10	9.0	0.0	(0.0; 13.8)	69%	11.2	5.2	(0.0; 18.2)	56%	0.008	13.5	6.6	(0.0; 21.1)	57%	5.8	0.0	(0.0; 7.3)	81%	< 0.0001

		Subgroups by educational level^a^													Subgroup by overweight status^a^								
		Low				Intermediate				High				*p* value^b^	BMI < 25 kg/m^2^				BMI ≥ 25 kg/m^2^				*p* value
		Mean	Median	(P25; P75)	%adh	Mean	Median	(P25; P75)	%adh	Mean	Median	(P25; P75)	%adh		Mean	Median	(P25; P75)	%adh	Mean	Median	(P25; P75)	%adh	
Denmark		(*n* = 248)				(*n* = 943)				(*n* = 548)					(*n* = 972)				(*n* = 739)				
Fruit, g/day	≥ 200	152	94	(0.0; 234)	29%	159	115	(30.4; 233)	32%	214	167	(64; 305)	42%	< 0.0001	167	124	(33.1; 246)	34%	174	129	(23.5; 255)	33%	0.382
Vegetables, g/day	≥ 200	126	96	(56; 152)	16%	150	118	(63; 185)	21%	184	137	(84; 238)	32%	< 0.0001	154	118	(66; 191)	23%	146	108	(63; 182)	21%	0.072
Legumes, g/day	≥ 19	6.1	0.4	(0.0; 6.7)	10%	6.5	1.6	(0.0; 6.8)	10%	7.7	2.8	(0.0; 7.8)	11%	< 0.0001	6.4	1.9	(0.0; 6.9)	9%	6.9	1.5	(0.0; 7.4)	11%	0.055
Red and processed meat, g/day	≤ 71	102	90	(58; 143)	39%	99	92	(58; 131)	33%	82	75	(44.5; 111)	46%	< 0.0001	94	86	(52; 126)	38%	99	90	(54; 134)	37%	0.072
Alcohol, g/day	≤ 10	13.2	6.3	(0.0; 21.4)	58%	13.7	6.0	(0.0; 20.6)	59%	15.0	8.8	(0.0; 24.5)	52%	0.226	13.2	6.2	(0.0; 20.5)	58%	14.5	6.7	(0.0; 23.4)	57%	0.100
Czech Republic		(*n* = 345)				(*n* = 1194)				(*n* = 127)					(*n* = 802)				(*n* = 864)				
Fruit, g/day	≥ 200	89	61	(1.3; 141)	11%	122	82	(13.4; 173)	21%	121	96	(40.1; 179)	20%	0.0004	112	79	(19.1; 165)	19%	118	79	(5.9; 168)	19%	0.371
Vegetables, g/day	≥ 200	90	71	(40.0; 123)	8%	94	74	(37.0; 126)	10%	120	85	(59; 160)	15%	0.002	96	77	(40.0; 126)	10%	95	73	(37.8; 128)	9%	0.807
Legumes, g/day	≥ 19	8.9	0.0	(0.0; 3.0)	12%	7.3	0.0	(0.0; 2.0)	11%	7.3	0.0	(0.0; 2.7)	11%	0.524	7.3	0.0	(0.0; 2.3)	11%	7.9	0.0	(0.0; 2.1)	11%	0.588
Red and processed meat, g/day	≤ 71	96	86	(47.4; 134)	42%	88	82	(44.3; 124)	41%	81	72	(43.4; 117)	48%	0.035	83	73	(40.0; 121)	48%	94	88	(50.2; 130)	37%	0.0002
Alcohol, g/day	≤ 10	11.7	5.0	(0.0; 19.0)	61%	10.5	4.8	(0.0; 16.3)	66%	10.1	7.7	(0.0; 16.8)	61%	0.354	10.4	4.5	(0.0; 16.9)	65%	11.0	5.5	(0.0; 16.9)	64%	0.402
Italy		(*n* = 692)				(*n* = 985)				(*n* = 507)					(*n* = 1484)				(*n* = 828)				
Fruit, g/day	≥ 200	182	155	(69; 260)	38%	183	149	(65; 250)	36%	206	169	(83; 282)	41%	0.027	185	155	(68; 249)	37%	187	150	(68; 272)	37%	0.788
Vegetables, g/day	≥ 200	242	206	(137; 296)	53%	238	205	(136; 300)	52%	232	202	(129; 287)	51%	0.534	229	200	(130; 288)	50%	254	213	(144; 323)	55%	0.0001
Legumes, g/day	≥ 19	11.7	0.0	(0.0; 4.1)	22%	10.7	0.0	(0.0; 3.3)	19%	10.1	0.0	(0.0; 4.5)	17%	0.560	10.5	0.0	(0.0; 2.3)	19%	11.1	0.0	(0.0; 4.2)	19%	0.592
Red and processed meat, g/day	≤ 71	88	81	(41.0; 122)	65%	85	77	(37.5; 119)	65%	83	77	(35.9; 121)	65%	0.332	84	77	(36.8; 118)	65%	86	78	(39.2; 124)	64%	0.433
Alcohol, g/day	≤ 10	8.8	0.0	(0.0; 15.3)	66%	7.1	0.1	(0.0; 11.9)	72%	7.4	0.2	(0.0; 11.1)	74%	0.001	6.8	0.0	(0.0; 11.2)	73%	9.6	4.0	(0.0; 15.9)	62%	< 0.0001
France		(*n* = 1039)				(*n* = 495)				(*n* = 737)					(*n* = 1379)				(*n* = 871)				
Fruit, g/day	≥ 200	125	76	(0.0; 200)	24%	128	84	(0.0; 195)	21%	137	95	(13.9; 196)	23%	0.265	126	82	(0.0; 191)	22%	134	89	(0.0; 204)	24%	0.180
Vegetables, g/day	≥ 200	181	152	(77; 248)	36%	179	144	(74; 245)	33%	183	156	(87; 249)	37%	0.892	175	146	(75; 242)	33%	188	158	(85; 254)	39%	0.036
Legumes, g/day	≥ 19	19.5	0.0	(0.0; 1.3)	21%	13.2	0.0	(0.0; 0.4)	15%	12.5	0.0	(0.0; 0.5)	15%	0.0003	16.3	0.0	(0.0; 1.1)	19%	15.5	0.0	(0.0; 0.5)	16%	0.645
Red and processed meat, g/day	≤ 71	102	91	(48.7; 144)	39%	90	79	(33.5; 129)	44%	84	74	(33.9; 123)	47%	< 0.0001	89	78	(35.7; 127)	44%	101	91	(48.7; 145)	40%	0.0001
Alcohol, g/day	≤ 10	8.3	0.0	(0.0; 11.8)	73%	9.4	0.2	(0.0; 15.1)	66%	9.6	0.2	(0.0; 15.5)	67%	0.135	8.0	0.0	(0.0; 12.1)	73%	10.6	0.1	(0.0; 16.9)	64%	< 0.0001

Intake of food groups are standardised to a 2000 kcal/day diet

%adherence represents a proxy for the percentage of the population that adhere to food-based dietary guidelines

*BMI* Body Mass Index

^a^Younger and middle-aged adults, aged 18–64 years, were stratified by gender, educational level and overweight status

^b^*p* value for the overall comparisons between population subgroups

### Foods to increase

Mean fruit and vegetable intake varied significantly between countries with lower intakes for Czech Republic (118 and 95 g/day, respectively) and higher intakes for Italy (199 and 239 g/day, respectively), and varied in the same direction between men and women within all four countries showing higher intakes for women. Higher fruit intake was also observed in all four countries for the elderly and for subjects with a higher educational level, but no differences by overweight status. Vegetable intake tended to be higher among elderly in Denmark and France, among higher educated subjects in Denmark and Czech Republic, and among overweight subjects in Italy and France. Mean intakes of legumes (6.5–16.7 g/day), and nuts and seeds (0.5–2.6 g/day) were generally low in all countries. Mean intake of dairy was higher in Denmark (302 g/day), while fish was higher in Italy (44.6 g/day) and France (34.4 g/day).

### Foods to decrease

Mean intake of red and processed meat was generally high in all countries (84–94 g/day). Within-countries, red and processed meat intake was lower for the elderly and women in all four countries, and except in Italy for the higher educated subjects, and in Czech Republic and France for the non-overweight. Alcohol intake varied between countries with lower intakes in Italy (8.2 g/day) and higher intakes for Denmark (14.6 g/day), and varied within countries in the same direction by gender and overweight status with lower intakes for women and the non-overweight. Alcohol intake also tended to be lower for the young and middle-aged adults, except in Czech Republic where intake is lower for the elderly. For the higher-educated subjects, alcohol intake tended to be lower in Czech Republic and Italy, but higher in Denmark and France.

### Foods to replace

Mean intakes of whole grains from cereals, pasta and bread were low in all countries, illustrated by the fraction of whole grains on total grains of ≤ 15% with one exception for wholegrain pasta in France. Although mean intake of total breakfast cereals per day was very low, the whole grain variants were primarily eaten. Intake of white meat was much lower than red and processed meat, in particular red and processed meat contributed to 70–80% of total meat intake comprising mainly of red meat in Denmark, Italy and France, and of processed meat in Czech Republic. Intakes of butter and hard margarines were only slightly higher than intakes of soft margarines and vegetable oils, except for Denmark where butter and hard margarines were predominantly chosen as fat source, and for Italy where vegetable oils were dominating.

## Nutrients

Table 4 shows the energy-standardised nutrient intakes, their corresponding proxy prevalence figures for inadequate intakes, and the NRD scores in four European adult populations, aged ≥ 18 years. Low intakes were observed for dietary fibre (15.8–19.4 g/day) and vitamin D (2.4–3.0 µg/day) in all countries, and for potassium (2288–2939 mg/day), and magnesium (268–285 mg/day), except in Denmark. Intake of vitamin E was lower in Denmark (6.7 mg/day), and folate in Czech Republic (212 µg/day). Mean intakes were high for protein (67.1–83.5 g/day), and iron (9.1–12.4 mg/day) in all countries analysed. Remaining nutrients, including calcium, zinc, vitamin A, C, B1, B2, and B12, showed varying intake levels between countries. Of the three nutrients to limit, a large penalty was obtained from saturated fatty acids (11.1–15.1 E%) in all countries, and from estimated sodium intake (2797–4244 mg/day) except in Italy. Based on the NRD scores, it is apparent that the nutrient density of the diet was highest in Italy (NRD9.3 of 537, and NRD15.3 of 1051), followed by Denmark (NRD9.3 of 416, and NRD15.3 of 896) and France, and the lowest in Czech Republic (NRD9.3 of 327 and NRD15.3 of 787). Within countries, nutrient density of the diet tended to be higher for women in all four countries and for the higher-educated subject, except in Italy (Table 5).

**Table 4:** Energy-standardised nutrient intakes, prevalence of inadequate intake, and Nutrient Rich Diet scores in four European populations, aged ≥ 18 years

	DRV	Denmark (*n* = 2025)				Czech Republic (*n* = 1869)				Italy (*n* = 2831)				France (*n* = 2624)			
		Mean	Median	(P25; P75)	%<DRV	Mean	Median	(P25; P75)	%<DRV	Mean	Median	(P25; P75)	%<DRV	Mean	Median	(P25; P75)	%<DRV
Unstandardised energy intake, kcal/day	–	2264*	2155	(1681; 2738)	–	2523*	2396	(1790; 3106)	–	2119*	2057	(1666; 2491)	–	1980*	1912	(1509; 2390)	–
Qualifying nutrients																	
Protein, g/day	0.66 g/BW	68.7	67.6	(59.7; 77.1)	16%	67.1	66.1	(59.1; 73.8)	12%	79.0*	77.8	(70.5; 86.1)	1%	83.5*	81.4	(70.9; 93.4)	2.4%
Protein, E%	–	13.9	13.8	(12.4; 15.2)	–	13.4	13.2	(11.8; 14.8)	–	15.6*	15.6	(14.1; 17.2)		16.7*	16.3	(14.2; 18.7)	
Animal protein, g/day	–	44.8*	43.2	(35.6; 52.8)	–	38.8*	37.5	(30.1; 45.8)	–	48.6*	47.1	(38.9; 56.8)	–	^c^			
Plant protein, g/day	–	20.3*	20.2	(16.9; 23.6)	–	23.9*	23.8	(20.1; 27.3)	–	30.3*	30.3	(26.5; 34)	–	^c^			
Dietary fibre, g/day^a^	25	19.4*	18.6	(14.5; 23.2)	81%	15.8*	15.1	(12.7; 18.3)	96%	18.1*	17.0	(14.0; 21.0)	88%	16.6*	15.7	(12.3; 19.5)	91%
MUFA, g/day^a^	–	25.7*	25.5	(21.0; 30.0)	–	32.0*	31.8	(27.8; 36.4)	–	39.0*	38.7	(33.5; 44.1)	–	29.7*	28.9	(24.0; 34.2)	–
MUFA, E%	10–20 E%	11.7*	11.6	(9.5; 13.6)	31%	14.4*	14.3	(12.5; 16.4)	8%	17.6*	17.4	(15.1; 19.9)	25%	13.4*	13.0	(10.8; 15.4)	23%
Calcium, mg/day	750	983*	928	(705; 1189)	30%	660*	593	(424; 805)	69%	742*	708	(539; 897)	57%	899*	842	(649; 1066)	38%
Iron, mg/day	M: 6; F: 7	9.1*	8.9	(7.7; 10.2)	8%	10.6*	10.1	(8.5; 12.1)	4%	11.1*	10.5	(9.0; 12.3)	2%	12.4*	11.2	(9.4; 13.8)	2%
Potassium, mg/day^b^	3500	3143*	3073	(2514; 3658)	69%	2288*	2199	(1895; 2573)	96%	2938	2834	(2420; 3326)	81%	2879	2763	(2326; 3287)	82%
Magnesium, mg/day^b^	M: 350; F: 300	322*	315	(270; 365)	54%	285	274	(241; 315)	75%	268*	254	(219 299)	80%	282	263	(230 ; 309)	77%
Zinc, mg/day	M: 7.5; F: 6.2	9.5*	9.3	(8.1; 10.8)	10%	7.0*	6.7	(5.6; 8.0)	52%	11.0*	10.5	(9.1; 12.4)	3%	10.2*	9.6	(8.1; 11.8)	9%
Vitamin A, µg RE/day	M: 570; F490	1032*	851	(557; 1242)	23%	692*	450	(315; 631)	62%	854*	635	(467; 924)	34%	1200*	822	(552; 1279)	23%
Vitamin C, mg/day	M: 90; F: 80	102*	85	(57; 131)	50%	78*	63	(37; 103)	65%	126*	103	(66; 159)	38%	91*	76	(46; 119)	56%
Vitamin E, mg/day^b^	M: 13; F: 11	6.7*	6.1	(5.1; 7.7)	95%	11.7*	11.1	(8.4; 14.4)	56%	12.7*	11.8	(9.7; 14.1)	53%	10.6*	9.4	(6.9; 13.2)	66%
Vitamin D, µg/day^b^	15	3.0	1.9	(1.3; 2.7)	97%	2.9	2.1	(1.4; 3.2)	99%	2.4	1.5	(1.0; 2.4)	99%	2.6	1.7	(1.0; 3.0)	99%
Vitamin B1, mg/day	0.6	1.1	1.1	(0.9; 1.3)	3%	1.1	1.0	(0.9; 1.2)	2%	1.10	0.9	(0.8; 1.1)	53%	1.20	1.1	(0.9; 1.3)	0%
Vitamin B2, mg/day	M: 1.1; F: 0.9	1.47*	1.38	(1.13; 1.70)	20%	1.08*	0.99	(0.84; 1.20)	65%	1.40*	1.3	(1.1; 1.6)	16%	1.80*	1.7	(1.4; 2.1)	8%
Vitamin B12, µg/day^b^	4	4.7	4.2	(3.1; 5.6)	45%	4.4	3.4	(2.5; 4.8)	64%	6.1	4.1	(3.1; 5.8)	48%	5.6	4.0	(2.9; 5.8)	50%
Folate, µg DFE/d	250	293	268	(214; 334)	41%	212*	182	(146; 242)	76%	350*	305	(254; 380)	23%	278	253	(203; 322)	49%
Disqualifying nutrients	MRV				%> MRV				%> MRV				%> MRV				%> MRV
SFA, g/day	–	30.4	30.2	(25.0; 35.4)	–	30.6	30.4	(25.5; 35.1)	–	24.6*	24.2	(20.3; 28.3)	–	33.5*	33.4	(27.7; 39.1)	–
SFA, E%/day^d^	< 10 E%	13.8	13.7	(11.3; 16.1)	86%	13.8	13.7	(11.5; 15.8)	80%	11.1*	10.9	(9.1; 12.7)	62%	15.1*	15.0	(12.5; 17.6)	91%
Added sugar, g/day	–	43.2*	36.4	(21.3; 57.2)	–	36.6	31.3	(18.8; 50.6)	–	38.6	35.2	(21.1; 52.5)	–	^c^			–
Added sugar, E%^d^	< 10 E%	8.8*	7.4	(4.3; 11.6)	32%	7.3	6.3	(3.8; 10.1)	21%	7.7	7.0	(4.2; 10.5)	24%	^c^			^b^
Sodium, mg/day^d^	< 2400	3012*	2919	(2484; 3439)	80%	4244*	4153	(3576; 4800)	98%	1703*	1648	(1245; 2076)	13%	2797*	2668	(2228; 3223)	85%
Nutrient Rich Diet Scores																	
Sub-score NRD9	–	765	775	(710; 829)	–	715*	721	(643; 794)	–	781*	793	(730; 841)	–	759	767	(701; 826)	–
Sub-score NRD15	–	1245	1259	(1192; 1310)	–	1175*	1182	(1097; 263)	–	1295*	1310	(1246; 1356)	–	1250	1262	(1191; 1324)	–
Sub-score NRDX.3	–	349*	346	(300; 392)	–	388*	387	(347; 427)	–	244*	242	(215; 271)	–	^c^			–
Total score NRD9.3	–	416*	427	(334; 507)	–	327*	328	(256; 400)	–	537*	547	(482; 600)	–	^c^			–
Total score NRD15.3	–	896*	916	(823; 992)	–	787*	791	(704; 875)	–	1051*	1062	997; 1115	–	^c^			–

*DRV* dietary reference value, *AR* average requirement, *AI* adequate intake, *RE* retinol equivalents, *DFE* dietary folate equivalents, *E%* energy percentage, *MUFA* mono-unsaturated fatty acids, *SFA* saturated fatty acids, *NRD* Nutrient Rich Diet scores, including their sub-scores

Intakes of nutrients are standardised to a 2000 kcal/day diet

^a^%<AR represents a proxy for the percentage of the population that have an inadequate intake, i.e. intake lower than the dietary reference value

^b^Nutrients where AR cannot be set, hence AI is defined

^c^Cannot be computed

^d^Percentages shown for SFA, added sugar and sodium reflect the proportion of the population that have an excessive intake, i.e. intake higher than the reference value (Maximum Recommend Value)

*Bonferroni *p* < 0.0001 test comparison for intake that was significantly different from all other three countries under study

**Table 5:** Nutrient density of the diet, using Nutrient Rich Diet scores 9.3 and 15.3, in four European populations in subgroups by age, gender, educational level and overweight status

	Subgroups by age							Subgroups by gender^a^						
	Younger and middle-aged adults			Elderly,≥ 65 years			*p* value	Men			Women			*p* value
	Mean	Median	(P25; P75)	Mean	Median	(P25; P75)		Mean	Median	(P25; P75)	Mean	Median	(P25; P75)	
Denmark	(*n* = 1739)			(*n* = 286)				(*n* = 777)			(*n* = 965)			
Sub-score NRD9	764	774	(708; 829)	772	787	(721; 833)	0.120	731	733	(679; 786)	796	808	(758; 853)	< 0.0001
Sub-score NRD15	1243	1256	(1191; 1308)	1256	1275	(1198; 1325)	0.033	1215	1227	(1162; 1280)	1271	1284	(1226; 1328)	< 0.0001
Sub-score NRDX.3	351	348	(301; 395)	333	336	(291; 382)	< 0.0001	355	353	(309; 400)	346	339	(297; 388)	0.011
Total score NRD9.3	413	424	(327; 505)	439	424	(328; 505)	0.001	376	386	(295; 456)	450	465	(388; 537)	< 0.0001
Total score NRD15.3	892	913	(817; 988)	923	940	(847; 1010)	0.003	860	876	(780; 944)	925	944	(859; 1021)	< 0.0001
Czech Republic	(*n* = 1666)			(*n* = 203)				(*n* = 793)			(*n* = 873)			
Sub-score NRD9	714	720	(641; 793)	729	728	(666; 807)	0.037	659	656	(597; 719)	763	777	(713; 821)	< 0.0001
Sub-score NRD15	1174	1182	(1092; 1261)	1185	1181	(1114; 1269)	0.208	1119	1115	(1039; 1197)	1223	1235	(1157; 1297)	< 0.0001
Sub-score NRDX.3	387	385	(345; 427)	396	395	(360; 430)	0.053	375	377	(333; 417)	398	397	(358; 436)	< 0.0001
Total score NRD9.3	327	327	(253; 400)	333	342	(270; 401)	0.456	284	283	(216; 349)	366	373	(298; 440)	< 0.0001
Total score NRD15.3	787	790	(703; 876)	789	792	(711; 873)	0.830	744	744	(665; 821)	826	836	(751; 910)	< 0.0001
Italy	(*n* = 2313)			(*n* = 518)				(*n* = 1068)			(*n* = 1245)			
Sub-score NRD9	777	790	(725; 837)	796	805	(759; 852)	< 0.0001	747	754	(692; 806)	803	814	(764; 856)	< 0.0001
Sub-score NRD15	1293	1307	(1240; 1350)	1305	1321	(1272; 1360)	0.003	1264	1271	(1210; 1330)	1317	1329	(1278; 1367)	< 0.0001
Sub-score NRDX.3	245	243	(215; 271)	242	240	(213; 269)	0.464	242	240	(212; 271)	247	245	(219; 272)	0.004
Total score NRD9.3	533	541	(476; 598)	554	563	(509; 609)	< 0.0001	505	513	(443; 572)	556	565	(509; 614)	< 0.0001
Total score NRD15.3	1048	1059	(991; 1115)	1064	1075	(1021; 1122)	0.002	1022	1032	(959; 1091)	1070	1079	(1024; 1127)	< 0.0001
France	(*n* = 2276)			(*n* = 348)				(*n* = 936)			(*n* = 1340)			
Sub-score NRD9	754	762	(696; 821)	785	787	(743; 841)	< 0.0001	717	723	(668; 775)	788	799	(743; 846)	< 0.0001
Sub-score NRD15	1244	1256	(1182; 1319)	1278	1289	(1222; 1346)	< 0.0001	1208	1219	(1147; 1284)	1278	1289	(1228; 1346)	< 0.0001

	Subgroups by educational level^a^										Subgroup by overweight status^a^						*p* value
	Low			Intermediate			High			*p* value^b^	BMI < 25 *kg*/*m*^*2*^			BMI ≥ 25 *kg*/*m*^*2*^			
	Mean	Median	(P25; P75)	Mean	Median	(P25; P75)	Mean	Median	(P25; P75)		Mean	Median	(P25; P75)	Mean	Median	(P25; P75)	
Denmark	(*n* = 248)			(*n* = 943)			(*n* = 548)				(*n* = 972)			(*n* = 739)			
Sub-score NRD9	746	754	(690; 814)	760	767	(705; 826)	791	803	(743; 844)	< 0.0001	769	779	(717; 829)	759	766	(702; 831)	0.054
Sub-score NRD15	1221	1236	(1165; 1293)	1242	1254	(1193; 1306)	1271	1282	(1224; 1325)	< 0.0001	1250	1261	(1204; 1308)	1237	1249	(1177; 1309)	0.021
Sub-score NRDX.3	356	356	(305; 404)	356	350	(304; 401)	334	334	(291; 370)	<0.0001	351	349	(305:392)	351	347	(295; 398)	1.000
Total score NRD9.3	390	404	(292; 498)	405	414	(324; 492)	456	459	(392; 537)	< 0.0001	408	418	(316; 511)	408	418	(316; 511)	0.2448
Total score NRD15.3	865	893	(767; 978)	887	905	(817; 978)	937	942	(869; 1013)	< 0.0001	887	908	(791; 990)	887	907	(791; 990)	0.165
Czech Republic	(*n* = 345)			(*n* = 1194)			(*n* = 127)				(*n* = 802)			(*n* = 864)			
Sub-score NRD9	695	684	(624; 780)	716	722	(644; 794)	740	744	(682; 802)	< 0.0001	719	725	(646; 795)	709	713	(633; 791)	0.036
Sub-score NRD15	1153	1149	(1060; 1252)	1175	1181	(1098; 1259)	1217	1238	(1149; 1281)	< 0.0001	1175	1186	(1097; 1260)	1172	1178	(1091; 1261)	0.605
Sub-score NRDX.3	378	378	(339; 421)	390	387	(346; 430)	384	381	(348; 413)	0.007	389	390	(347; 430)	385	382	(343; 424)	0.196
Total score NRD9.3	317	307	(237; 387)	327	327	(254; 406)	356	360	(301; 403)	0.003	330	329	(258; 400)	324	323	(248; 399)	0.260
Total score NRD15.3	775	775	(681; 862)	785	789	(706; 874)	833	847	(771; 904)	< 0.0001	786	791	(704; 876)	787	789	(703; 877)	0.872
Italy	(*n* = 692)			(*n* = 985)			(*n* = 507)				(*n* = 1484)			(*n* = 828)			
Sub-score NRD9	774	788	(718; 835)	776	789	(725; 834)	788	801	(734; 851)	0.005	779	792	(728; 838)	775	788	(720; 836)	0.245
Sub-score NRD15	1291	1309	(1234; 1355)	1292	1304	(1242; 1353)	1300	1316	(1249; 1360)	0.140	1294	1308	(1244; 1355)	1291	1307	(1234; 1354)	0.414
Sub-score NRDX.3	240	240	(211; 267)	246	243	(217; 273)	249	246	(220; 276)	0.001	248	245	(219; 273)	240	237	(209; 268)	< 0.0001
Total score NRD9.3	534	545	(478; 603)	530	536	(474; 593)	539	550	(480; 603)	0.158	531	539	(475; 598)	535	545	(476; 597)	0.289
Total score NRD15.3	1051	1065	(992; 1118)	1046	1056	(993; 1111)	1051	1064	(991; 1115)	0.439	1046	1058	(992; 1114)	1051	1064	(990; 1115)	0.206
France	(*n* = 1039)			(*n* = 495)			(*n* = 737)				(*n* = 1379)			(*n* = 871)			
Sub-score NRD9	749	760	(681; 822)	756	763	(702; 817)	761	764	(707; 825)	0.014	753	760	(696; 819)	758	766	(699; 827)	0.181
Sub-score NRD15	1237	1252	(1166; 1319)	1247	1250	(1194; 1314)	1254	162	(1190; 1326)	0.002	1242	1256	(1177; 1316)	1249	1258	(1191; 1329)	0.110

*BMI* Body Mass Index, *NRD* Nutrient Rich Diet scores, including their sub-scores

For France, sub-score NRDX.3, total score NRD9.3 and 15.5 cannot be computed due to a lack of data on sugars

^a^Younger and middle-aged adults, aged 18–64 years, were stratified by gender, educational level and overweight status

^b^*p* value for the overall comparisons between population subgroups

## Discussion

In this study, we found that dietary intakes varied markedly across the four European countries, irrespective of energy intake. Within countries, food intakes also varied markedly by socio-economic factors such as age, gender, and educational level, but less pronounced by anthropometric factors such as overweight status. However, the set of food-based dietary guideline was not met by a large part of the population and/or population subgroup by age, gender, educational level or overweight status.

When describing food group intakes, mean daily intakes of fruit and vegetables, sweet beverages, and alcohol varied most between countries, showing higher intakes of fruit and vegetables, and lower intakes of sweet beverages and alcohol in Italy. In addition, we observed in Italy and France a similar vegetable intake among the different levels of education, whereas in Denmark and Czech Republic higher intake of vegetables was observed among higher-educated subjects; which is in line with previous studies conducted in European populations [40–42]. This region-dependent tendency might be attributed to the long-standing cultural tradition of using vegetables in the Mediterranean diet, as consumed in Italy and France, and is often easily recognisable by all layers of the population. However, a comparison of population subgroups within-countries is often closely related to dietary preferences, beliefs and practices of that particular consumer group. Higher intake of fish, nuts and seeds along with lower intake of red and processed meat are, for example, generally seen among women and higher-educated subjects, which might be driven by their health considerations and awareness of climate change [43].

When describing nutrient intakes summarised by the NRD9.3 and 15.3, the higher scores were observed for Italy, which is mainly attributed to their lower penalty score, i.e. NRDX.3, for the disqualifying nutrients of SFA and sodium. Because of the interrelation between food groups and nutrients intake, our results on variation in nutrient intakes can be partly reflected by our results on variation in food group intake. Low penalty score in Italy is likely to be in correspondence with its lower intakes for important sources of SFA intake such as butter and hard margarines, red and processed meat, and dairy products; however, with the estimates of sodium intake, caution must be applied, as they are very likely to be under-estimated due to difficulties in quantifying sodium content in recipes and discretionary salt intake [44]. Moreover, when focussing on qualifying nutrients, higher sub-scores NRD9 and NRD15 were also observed for Italy, but intake for calcium, potassium and magnesium was lower when compared with Denmark; related to intake of dairy products and whole-grain products. It could, thus, be argued whether these summary estimates could be used solely to describe nutrient intakes, as they do not point out specific inadequate nutrient intakes.

In the context of the SUSFANS project, we prefer to describe dietary intakes in terms of foods rather than nutrients, since foods are the constituents of a dietary pattern and the common denominator for linking dietary intakes with health, environment, affordability, consumer’s preferences, etc. Diet-associated environmental impact, in particular, has been attracting a lot of interest, as current food production and consumption patterns have been recognised as a major human-induced driver of climate change [45]. Some European countries have, therefore, developed guidelines for diets that are both healthy and environmentally-friendly [46–49]. Such recommendations mostly emphasise the reduction of greenhouse gas emissions through propagating a shift towards plant-based foods. However, given European dietary intakes, there is still much progress to be made in this respect, simply showed by a percentage of around 35% for the intake of plant protein as opposed to total protein for the countries we studied. Moreover, predominant food groups contributing to animal and plant protein intake have been associated with regional and cultural traditions around dietary habits. Meat intake is regarded as the most important contributor to animal protein in European diets, but with differences related to the amount and types of meat consumed, as also denoted by previous studies [50, 51]. With regard to plant protein, cereals and cereal products have been identified as the main contributor to plant protein in European diets [52], while joint contributions from vegetables, legumes and fruit varied between countries, as observed in the present study.

The present study provides further support for the application of individual-level dietary data to address the food-climate connection. Often diet-associated environmental impact was quantified using food availability data related to food production, but not to food consumption as such. Using individual-level reported dietary data might, therefore, be regarded as a useful tool in the connection between health and environment with foods as their common denominator. Cross-country comparison of individual-level dietary data is, however, challenged by the dietary surveys conducted with different survey characteristics and data collection methods that may influence the comparability of the results. First, sampling procedures used in the surveys reported in this study varied in terms of recruitment methods, household and individual representativeness, number of subjects per household and weighting factors used; however, they all aimed at including a nationally representative sample of at least all age-sex categories. It still remains a possibility that those who have agreed to participate form a group with a greater interest in health, hence more optimistic results.

Second, methods of dietary assessment used in the surveys reported were conducted differently, with regard to the methods used and in the manner in which the assessment was carried out. Replicates of 24-h recall as applied in Czech Republic showed a higher mean energy intake compared to diet records as applied in Denmark, Italy and France. This might be explained by factors related to the methods themselves, such as reliance on memory and portion size estimations [53–55], and/or characteristics of the populations. Standardising intake data to a 2000 kcal/day diet had, therefore, the largest impact on results of Czech Republic; lowering its mean dietary intakes under the assumption that energy intake is positively correlated with food group and nutrient intake. Standardisation for energy is one of the more practical ways of reducing part of the extraneous variation in dietary estimates [56], and enables to study the relative contribution of food groups and nutrients intake to the total diet, regardless of energy intake. In the European Food COnsumption VALidation project, it has been suggested to adjust for BMI instead when analysing and interpreting dietary data of nutritional monitoring surveys to reduce mean bias at population level [57]. Given that stratified analyses by overweight status showed no relevant differences in dietary intakes within a country, it is questionable whether BMI-adjusted values should be the main exposure of interest in the present study describing the heterogeneity of European diets.

Another important factor in estimating dietary intakes consistently is the number of days included in the dietary assessment to enable comparison between countries across Europe. In this study, dietary data were, therefore, standardised for the number of days, but have not been corrected for time-interval between the two selected record/recall days, hence not corrected for within-subject day-to-day variability. Correcting for within-subject day-to-day variability would have resulted in comparable means for dietary intakes compared to unadjusted data, though with a shrinkage of intake distributions which in turn would have decreased the percentage of the population above and below a cut-off point [58]. However, relying on consecutive days, including days spaced over a week time-interval, is likely to underestimate the within-subject day-to-day variation [59] because of the interdependence of days that captures some of the day-to-day variation in the between-subject variation [60, 61]. Thus, this day-interdependence would have resulted in a shrinkage of the observed intake distribution that is too much toward the group mean, hence an under-estimation of true percentage of the population above and below a cut-off when statistically correcting intake distributions. In addition, the use of country-specific food composition databases might affect the number of subjects whose intake was below the DRV. In particular, when using different food composition databases, potential systematic errors in estimating nutrient intake would be different between countries, and in all probability alternate with magnitude and direction. With increasing globalisation, however, the foods and mixed dishes available in different countries are not all grown/produced/prepared in the same manner and, therefore, using a country-specific composition database is likely to reflect nutrient intake more accurately.

Exclusion of under-reporters would have increased the prevalence of adherence to the food-based dietary guidelines and decreased the prevalence of inadequate nutrient intakes, and inclusion of supplementation use would have decreased the prevalence of nutrient inadequacy even further. The present study did estimate the percentage of under- and over-reporters (Online Resource 1), but did not estimate intakes excluding them, because some of the mis-reporters may truly be consuming a low- or a high-energy diet. Over the past decades, dietary supplementation use has increased in Europe with a clear north–south gradient [62], showing a high number of users in Denmark (Online Resource 1). Hence, it is likely that in countries with higher level of supplementation use, dietary supplementation might have contributed to improved total nutrient intakes, with its impact dependent on the supplementation formulation, the frequency of use, and the level of micronutrient intakes of those taking supplements. However, our interest is on nutrient intakes from foods only to find nutritional gaps that are most in need to improve the healthiness of dietary intake.

In conclusion, there is considerable variation in food and nutrient intakes across European countries. The present study indicated that the intake of food groups showed larger deviations from food-based dietary guidelines for the overall population and population subgroups of the countries we studied. In addition, results suggested inadequate nutrient intakes from foods for dietary fibre and vitamin D in all countries, and for potassium, magnesium, vitamin E and folate in specific regions. Individual-level dietary data in different European population and population subgroups are, therefore, needed for balancing diets for European citizen.

Moreover, individual-level dietary data from national surveys serve as a practical tool for describing the healthiness of diet in terms of foods and nutrients, but dietary data harmonisation remains challenging. Using a common food classification system is a first step in the alignment of surveys and necessary to enable cross-country comparisons for food group intakes. However, further steps, such as standardisation for energy, number of days, etc., are needed for harmonisation of dietary data. Besides the healthiness of dietary intake, these dietary surveys might also be important in shaping optimised diets where other factors, such as environmental impact, affordability and consumer preferences are incorporated. We aim, therefore, to support further engagement of key stakeholders from the food supply chain and policy-makers in the next stages for the design of SHARP diets.

## Electronic supplementary material

Below is the link to the electronic supplementary material.

Supplementary material 1 (DOCX 13 KB)
