# Supplementary material for: Geographic and socioeconomic diversity of food and nutrient intakes: a comparison of four European countries
Source: Eur J Nutr. 2018 Mar 28;58(4):1475–93. doi: 10.1007/s00394-018-1673-6 (PMC6561990; doi:10.1007/s00394-018-1673-6)
Supplement: Supplementary file 1 — Supplementary material 1 (DOCX 13 KB) [file 394_2018_1673_MOESM1_ESM.docx]

**Online Resource 1** Percentage of under- and over-reporters as identified by Goldberg/Black equation, and percentage of dietary supplementation use in four European populations, aged ≥ 18 years.

|  | %under-reporters | | | %over-reporters | | | %supplement users | | |
| --- | --- | --- | --- | --- | --- | --- | --- | --- | --- |
|  | Total | Males | Females | Total | Males | Females | Total | Males | Females |
| Denmark | 17.7% | 18.2% | 17.3% | 1.4% | 1.3% | 1.5% | 60.5% | 55.0% | 65.9% |
| Czech Republic | 12.9% | 7.0% | 18.1% | 3.6% | 5.6% | 1.9% | 29.7% | 23.3% | 35.4% |
| Italy | 11.0% | 12.3% | 9.9% | 1.1% | 0.9% | 1.3% | 4.5% | 3.0% | 5.8% |
| France | 23.7% | 22.9% | 24.3% | 1.6% | 2.0% | 1.2% | 12.4% | 6.1% | 16.8% |
